# Radiofrequency Ablation of Gasserian Ganglion in Trigeminal Neuralgia With Multiple Sclerosis: A Rare Clinical Case

**DOI:** 10.7759/cureus.32595

**Published:** 2022-12-16

**Authors:** Vivek Chakole, Kapil Sharma, Jeshnu Tople, Shivani Akre, Mayur B Wanjari

**Affiliations:** 1 Department of Anaesthesiology, Jawaharlal Nehru Medical College, Datta Meghe Institute of Higher Education & Research (Deemed to be University), Wardha, IND; 2 Department of Medicine, Jawaharlal Nehru Medical College, Datta Meghe Institute of Higher Education & Research (Deemed to be University), Wardha, IND; 3 Department of Research and Development, Jawaharlal Nehru Medical College, Datta Meghe Institute of Higher Education & Research (Deemed to be University), Wardha, IND

**Keywords:** mandibular, trigeminal neuralgia, gasserian ganglion, antidepressants, drowsiness

## Abstract

In rare instances, the extremely painful disorder trigeminal neuralgia (TN) may develop as a result of multiple sclerosis (MS). In this article, we will be discussing the case of a 56-year-old female with TN. Antidepressants and analgesics can lessen the pain, although they were not very effective, and higher doses led to greater drowsiness and a poorer quality of life. Radiofrequency ablation helped this patient significantly lower the pain and led to an improved lifestyle. This case presents right-side radiofrequency ablation of Gasserian ganglion in a patient with unilateral TN with MS.

## Introduction

A neuropathic pain disorder affecting the trigeminal distribution of the face is termed trigeminal neuralgia (TN). Multiple sclerosis (MS) patients experience various neuropathic pain, with TN being the most severe. As per the International Association for the Study of Pain, TN is a sudden, typically unilateral, recurring pain that impacts one or more branches of the trigeminal nerve. TN pain might throb for anywhere between a few seconds or minutes, but when it is severe, it may persist for an hour. Starting in the pons, divisions of the trigeminal nerve are the ophthalmic (V1), maxillary (V2), and mandibular (V3) branches. The patient's physical capabilities and quality of life may be adversely impacted in TN. In a 2:1 ratio, women are more impacted than men. The nerve's maxillary and mandibular divisions are the most often impacted divisions [1-4]. Several treatment options include medicinal, surgical, and nerve block therapy [5]. TN can be a harrowing condition severely affecting the day-to-day activity of a patient. Here, we provide a successful treatment for TN in a patient with MS with radiofrequency ablation. The patient presented to our outpatient department with severe right-sided facial pain affecting her daily lifestyle.

## Case presentation

The main complaint of a 56-year-old female patient with known MS who visited our hospital was episodic discomfort across the right side of her face over the previous 12 months. Eating, swallowing, gritting her teeth, and cleaning her face all caused discomfort that was increased by light cutaneous or sensory stimulation. Her visual analog scale (VAS) rating for her neuropathic pain was a 9/10. She had been taking 300 mg carbamazepine tablet TDS daily and 20 mg baclofen tablet at night daily with 150mg pregabalin tablet at night for the previous two months. The patient complained of extreme drowsiness as a result of the higher medicine dosages.

The patient was medium built with a body mass index of 23, well-nourished, cooperative, and well-oriented to time, place, and person. The results of the systemic evaluation were normal. Upon closer inspection, it was found that the mandibular nerve region was heavily involved and that there was severe pain over its distribution. On ENT and dental opinion, which did not conclude any other diagnosis, we came to the diagnosis of TN in this patient with a known case of MS. 

**Figure 1:**
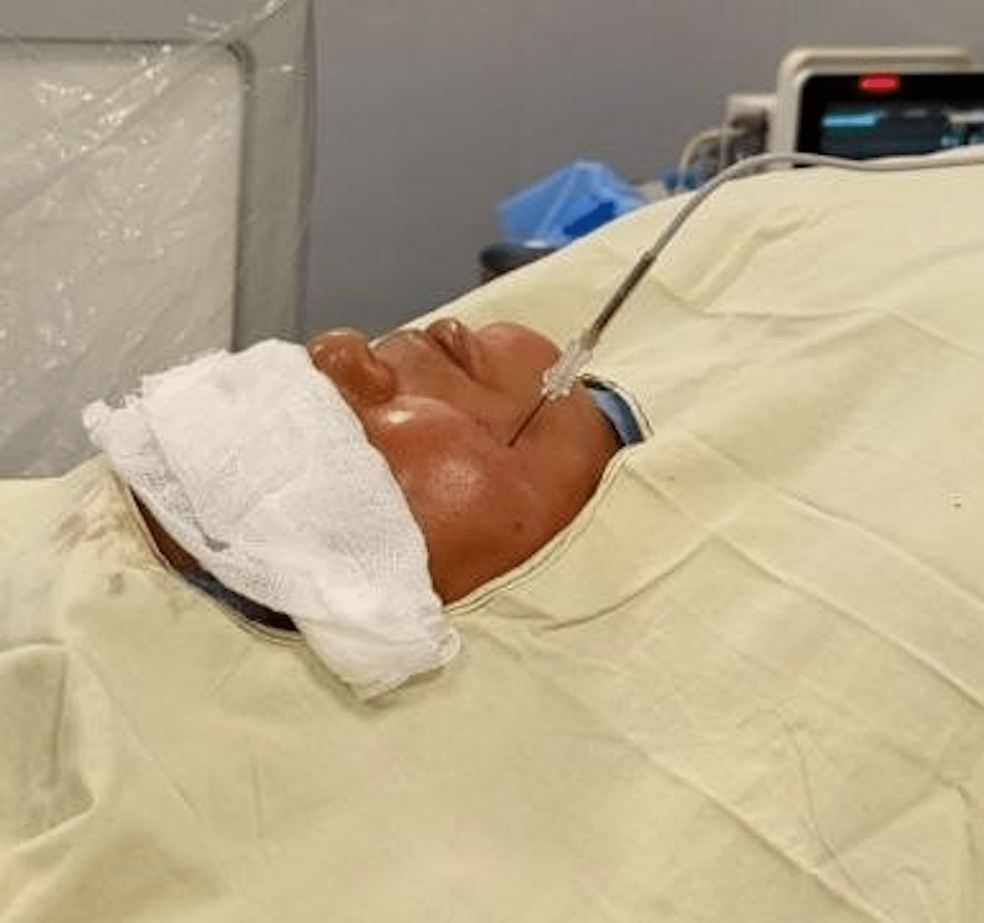
Right-Sided Radiofrequency Ablation of the Gasserian Ganglion

We prepared for the right-sided radiofrequency ablation of the Gasserian ganglion because the intensity of pain was high with harrowing in daily activity and also the patient refused medicinal therapy. Under all aseptic precautions and fluoroscopic guidance and local anesthesia, a 22G 10 cm cannula with an active tip of 5 mm was inserted. The cannula position was confirmed by a fluoroscope and by stimulation. A right-sided fluoroscopic-guided radiofrequency ablation was done at temperatures of 60° C, 65° C, and 70° C each for 90 seconds while the patient was lying on her back. With no difficulties, this led to decreased pain in the distribution of mandibular division. After six hours, the discomfort was effectively relieved. Only carbamazepine tablet, 200 mg twice daily, was given to the patient before discharge. The patient was doing well with day-to-day activities and had a VAS of 1/10 at the follow-up appointment after one month (Figure 1).

## Discussion

A disorder known as TN affects the fifth cranial nerve, which innervates the forehead, cheek, and lower jaw and has recurring, short bouts of electric shock-like pain. Although there is presently no curative treatment for TN, analgesics can be used to manage the discomfort. Whether these pain episodes are genuinely spontaneous attacks or are triggered by extremely modest sensory inputs or movements is still up for debate. Some patients experience warning symptoms like tingling before they experience pain. The trigeminal nerve provides motor and sensory supply to the masticatory muscles. Most frequently, TN presents unilaterally in patients affecting one side of the face [6-8]. Although rare, TN has the potential to leave some people with crippling neuropathic agony. Nerve blocks may be extremely important in treating patients who present with extremely painful illnesses because the main objective of treatment for these individuals is pain alleviation. The trigeminal nerve's anatomy and circulatory connections may be studied using magnetic resonance imaging, which efficiently identifies compression of the trigeminal nerve [6-9]. Multiple oral pharmacologic therapies (analgesics, muscle relaxants, etc.) are often used to alleviate pain in TN.

Demyelination, gliosis, and neuronal death are features of the chronic autoimmune disease multiple sclerosis of the central nervous system (CNS) [10]. Nerve fibers are protected by a material called myelin, which enables them to deliver quick electrical signals to the brain. In the case of MS, the immune system targets myelin and produces antibodies against it. Perivascular lymphocytic infiltrates and macrophages damage these myelin sheaths and lead to MS. Features of MS change depending on the location of the nerve involved. Neurological symptoms might include bladder and bowel incontinence, impairment of vision, tingling and numbness, and localized weakness. There are two primary pathological processes that make up the overall pathological process in MS patients, due to localized inflammation, the blood-brain barrier is harmed resulting in plaques and distinct CNS regions with microscopic damage due to neurodegeneration. Both macroscopic and microscopic harm is the consequence of these two main mechanisms working together [10-15].

In cases of MS, TN is one of the most common and challenging neuropathic painful conditions to treat. Daily routines, employment, mood, leisure time, and general quality of life can all be affected by TN in MS patients. According to research, a pontine demyelinating plaque is related to TN subsequent to MS. Although there are data to support the hypothesis that TN caused by MS may occasionally occur with neurovascular compression, there are presently no high-quality studies examining the effectiveness of microvascular decompression in individuals with MS [16-18]. The comparatively poorly tolerated medications with sedative and motor side effects as seen in this case underscore the need for the creation of new, more focused, and better-tolerated medications [16-18]. When something other than vascular compressions affects the trigeminal nerve's structural integrity, it is diagnosed as secondary trigeminal neuralgia, such as MS plaques, tumors, or anomalies of the skull base. TN is more likely to occur in people with MS [16]. TN symptoms often appear in waves and are followed by remission intervals. Some patients develop TN as a progressive disorder with shorter and shorter intervals between excruciating bouts [16]. Fifteen percent of MS patients present with TN which is difficult to diagnose [18].

In patients with TN, we can either give ganglionic block or do radiofrequency ablations. In Meckles Cave, the Gasserian Ganglion can be partly ablated using glycerol or by creating a radiofrequency lesion. Administering a Gasserian ganglion block requires technical proficiency and fluoroscopy assistance [19]. Gasserian ganglion block or radiofrequency ablation is performed when medical therapy is ineffective or when medical management results in negative effects or if the patient refuses to take medical management. There is a high risk of complications such as hypoesthesia, neuroparalytic keratitis, and weak masticatory muscles. In radiofrequency ablation, under local anesthesia, the cannula is put into the cheek and carefully guided by the use of a fluoroscope into Meckle's Cave, the place where the Gasserian Ganglion is located. To prevent excessive post-procedure numbness, it is crucial to treat the precise branch of the trigeminal nerve that is causing the discomfort [20-22]. Radiofrequency ablation is a quick, painless, and safe technique, but depending on the branch of the trigeminal nerve being used during the treatment, an appropriate temperature is required for ablation [23]. In our case given above, we did three cycles of ablation at 60° C, 65° C, and 70° C, each for 90 seconds. This ablation may assist those suffering from TN and other types of facial discomfort and will improve their quality of life.

## Conclusions

The most severe neuropathic pain that patients with MS might suffer is called trigeminal neuralgia. TN is one of the most challenging neuropathic conditions to treat. It can severely affect patients' lifestyles and can leave some patients with neuropathic agony. Radiofrequency ablation of Gasserian ganglion is performed when medicinal treatment is ineffective or in cases having side effects with medical management or if the patient is refusing to continue medical therapy. Radiofrequency ablation produces effective analgesia and decreases the doses of drugs needed for analgesia in the patient. Depending upon the division of the trigeminal nerve being ablated, different optimal temperatures are required for the radiofrequency ablation procedure. TN is a severely painful condition with no curative treatment available. For the management of pain, more study and advancement in neuropathic diseases and therapeutic techniques are crucial.
